# Photoinduced Inactivation of Pathogenic Microorganisms via Cotton Textile Functionalized with a Novel Iodinated  BODIPY Derivative

**DOI:** 10.3390/molecules31091525

**Published:** 2026-05-04

**Authors:** Awad I. Said, Desislava Staneva, William M. Piedra, Françisco M. Raymo, Ivo Grabchev

**Affiliations:** 1 Department of Chemistry, Faculty of Science, Assiut University, Assiut 71516, Egypt; 2 Frost Institute for Chemistry and Molecular Science, University of Miami, 1201 Memorial Drive, Coral Gables, FL 33146-2509, USA; wmp32@miami.edu (W.M.P.); fraymo@miami.edu (F.M.R.); 3 Department of Textile, Leather, and Fuels, University of Chemical Technology and Metallurgy, 1756 Sofia, Bulgaria; 4 Faculty of Medicine, Sofia University “St. Kliment Ohridski”, 1407 Sofia, Bulgaria

**Keywords:** BODIPY, photosensitizer, antimicrobial photodynamic inactivation, cotton textiles, singlet oxygen, bacteria, self-disinfecting surfaces

## Abstract

Antimicrobial resistance (AMR) is emerging as one of the most serious global health problems, necessitating the urgent development of alternative approaches to pathogen control. The present study describes the synthesis and characterization of a novel iodinated BODIPY derivative (BODIPY5), designed as a highly efficient photosensitizer for antimicrobial photodynamic inactivation (aPDI). The molecular design of the compound involves the introduction of two iodine atoms into the BODIPY5 core, which induces a “heavy atom effect”, accelerates the intersystem transition from the singlet to the triplet state, and leads to increased generation of singlet oxygen upon irradiation with visible light. Photophysical measurements show a significant fluorescence quenching of BODIPY5 compared to its unsubstituted counterpart, which is a direct indicator of increased photodynamic activity. The compound’s antimicrobial efficacy was tested in a homogeneous medium and after immobilization on cotton textiles via physical adsorption. In solution, BODIPY5 nearly eliminated the model bacterial strains *B. cereus* and *P. aeruginosa* at a low concentration of 10 µg/mL under light, with cell viability below 1%. The functionalized cotton fabric exhibits pronounced self-disinfection properties, retaining high photodynamic activity against the Gram-negative pathogen *P. aeruginosa*. Scanning electron microscopy results confirm extensive morphological damage and loss of structural integrity in bacterial cells on the treated textile following irradiation. The non-specific mechanism of action, which generates reactive oxygen species (^1^O_2_) in situ, prevents the development of bacterial resistance and makes the developed material a promising candidate for use in hospital environments, including antibacterial clothing and protective equipment.

## 1. Introduction

Antimicrobial resistance (AMR) is emerging as one of the most serious global health problems of our time, significantly limiting the effectiveness of conventional antimicrobial therapies. The uncontrolled and excessive use of antibiotics in humanitarian medicine, veterinary practice, and agriculture accelerates the emergence and spread of multidrug-resistant bacterial strains, such as “superbugs”, leading to increased morbidity, mortality and economic burden on healthcare systems worldwide [1,2]. This requires urgent research and development of non-antibiotic approaches with diverse strategies and mechanisms of action to control microorganisms. Antimicrobial textiles are emerging as an effective preventive measure to limit microbial contamination and pathogen spread, especially in hospital and public environments. Such materials are widely used in medical clothing, bed linen, curtains, personal protective equipment, sports and everyday textiles, as well as in the transport and interior sectors [3,4,5,6]. Among natural fibers, cotton occupies a leading position due to its biocompatibility, comfort, and biodegradability. At the same time, its hydrophilicity and porous structure, under certain conditions, create a favorable environment for the adhesion, proliferation, and formation of bacterial biofilms, which necessitates its functionalization with effective antimicrobial agents [7,8]. Traditional approaches to obtain antimicrobial textiles include the use of metallodendrimers, metal and metal oxide nanoparticles [9,10,11,12,13], quaternary ammonium salts [14,15], and various synthetic biocidal substances [16,17,18].

A promising approach is antimicrobial photodynamic inactivation (aPDI), which is based on the combined action of a photosensitizer, light of an appropriate wavelength, and molecular oxygen [19,20,21,22,23]. Upon photoactivation, the photosensitizer generates reactive oxygen species (ROSs), including singlet oxygen (^1^O_2_), which cause nonspecific oxidative damage to cell membranes, proteins, and nucleic acids of microorganisms [24,25,26,27,28]. Due to its multi-targeted mechanism of action, aPDI significantly reduces the likelihood of bacterial resistance. Among modern photosensitizers, BODIPY (boron-dipyrromethene) derivatives have attracted particular attention. They represent a class of organic fluorophores with excellent photophysical properties, including high molar absorption coefficients in the visible region, good photostability, and the ability to be precisely modified [29,30,31,32]. Initially, BODIPY compounds were used as fluorescent probes and labels in bioimaging, analytical chemistry, and sensing. However, in the last decade, they have also been established as effective photosensitizers for photodynamic applications, including antimicrobial photodynamic inactivation [33,34,35,36,37,38,39]. By introducing heavy atoms (e.g., bromine or iodine), cationic substituents, or π-extended structures, BODIPY derivatives can be optimized for increased intersystem crossing and efficient singlet oxygen generation [40,41,42,43].

The deposition of photosensitizers onto textile materials, including cotton fabrics, represents a promising approach for creating light-activatable antimicrobial textiles. This can be achieved through physical adsorption, polymer or sol–gel coatings, and covalent bonding to the cellulose chains of cotton [44,45].

The aim of the present work is to design and synthesize a new, highly effective BODIPY photosensitizer that, through precise structural modification with iodine atoms, enables powerful photodynamic inactivation of pathogenic microorganisms. The research aims to transform conventional cotton textiles into a “smart” self-disinfecting surface through non-covalent functionalization, combining the biocompatibility of natural fibers with light-induced antibacterial activity against multidrug-resistant strains. Through a comparative analysis of the photophysical properties and antimicrobial activity in a homogeneous environment and on a textile substrate, the work seeks to demonstrate the potential of this material to combat the global crisis of antibiotic resistance and prevent nosocomial infections.

## 2. Results and Discussion

### 2.1. Design of the Photosensitizer Diiodo BODIPY5

**Scheme 1:**
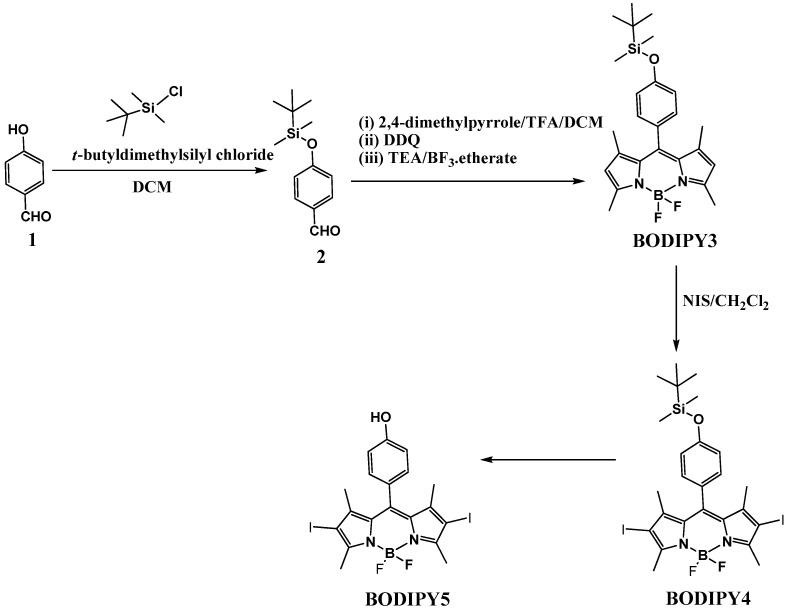
Steps in the synthesis of the photosensitizer BODIPY 5: (1) protection of the phenolic group of *p*-hydroxybenzaldehyde; (2) condensation with 2,4-dimethylpyrrole and subsequent oxidation and complexation to BODIPY3; (3) iodination to BODIPY4 and (4) removal of the protecting group to the final product BODIPY5.

BODIPY5 was designed and synthesized according to Scheme 1 using standard methods for modification of BODIPY derivatives [46,47]. Initially, the phenolic OH group of p-hydroxybenzaldehyde was protected by tert-butyldimethylsilyl chloride (TBDMS-Cl), to give compound 2. The protected aldehyde was then condensed with two equivalents of 2,4-dimethylpyrrole in dry dichloromethane (DCM) under an argon atmosphere, followed by oxidation with 2,3-dichloro-5,6-dicyano-1,4-benzoquinone (DDQ) and complexation with BF3·OEt2, which led to the formation of BODIPY3. BODIPY3 was iodinated with N-iodosuccinimide (NIS) in the dark to give BODIPY4. The final diiodo derivative BODIPY5 was obtained by deprotection of the silyl group by boiling in a DCM/CH_3_OH mixture in the presence of triethylamine (TEA). The resulting diiodo BODIPY possesses a phenolic OH group, which improves adsorption on cellulose fibers, and is capable of generating singlet oxygen under visible light. The introduction of iodine atoms into the BODIPY core leads to a “heavy atom effect” that accelerates intersystem crossing (ISC) from the singlet to the triplet excited state [40,48]. This leads to increased generation of singlet oxygen (^1^O_2_), which is key for the photodynamic inactivation of microorganisms. While unsubstituted BODIPY derivatives emit strong fluorescence, iodinated derivatives often exhibit characteristic fluorescence quenching, an indicator of efficient triplet-state formation and greater photodynamic activity. The chemical structures of the synthesized compounds were confirmed by ^1^H-NMR, ^13^C-NMR, and high-resolution mass spectrometry (HR-MS).

### 2.2. Photophysical Characteristics of BODIPY3, BODIPY4 and BODIPY5

**Figure 1:**
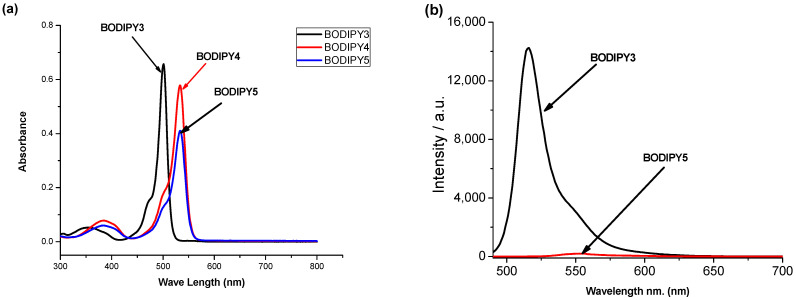
Absorption spectra of BODIPY 3–5 derivatives (**a**) and emission spectra of compounds BODIPY3 and BODIPY5 (**b**) in DMSO solution at concentration 10^−5^ M, λex. = 480 nm.

Furthermore, the wavelength of absorption, wavelength of emission, Stokes shift, absorption extinction coefficient, and quantum yield of BODIPY3, BODIPY4 and BODIPY5 in different solvents were determined (Table 1). The three dyes show typical BODIPY behavior with intense absorption in the visible region and relatively small Stokes shifts, but they differ markedly in fluorescence efficiency. BODIPY3 absorbs around 497–501 nm and emits at 516–521 nm with moderate Stokes shifts (≈690–766 cm^−1^), high molar extinction coefficients (up to 6.7 × 10^4^ M^−1^cm^−1^), and comparatively strong quantum yields (Φ_F_ = 0.30–0.52). In contrast, BODIPY4 and BODIPY5 are bathochromically shifted (λ_abs._ ≈ 528–535 nm; λ_fl_. ≈ 548–561 nm), consistent with extended conjugation induced by the electron pairs of iodine atoms, but they exhibit dramatically reduced quantum yields (Φ_F_ ≈ 0.009–0.022), despite still having high extinction coefficients due to the intersystem crossing (ISC) induced by iodine (heavy atom) [49], which is consistent with increased singlet oxygen generation and enhanced photodynamic activity, as shown in Figure 1.

### 2.3. Treatment of Cotton Fabric with BODIPY5

Following synthesis and spectral characterization, BODIPY5 was immobilized onto the cotton substrate via physical adsorption. The process utilized a fine emulsion technique (5 mg of BODIPY5 in 1 mL ethanol/20 mL H_2_O) at 50 °C, achieving a high loading efficiency of 88% (4.38 mg per gram of fabric). The phenolic -OH groups of the dye facilitate its attachment to the cellulose matrix through a synergistic combination of hydrogen bonding, van der Waals forces, and π–π interactions. Furthermore, the inherent water-insolubility of BODIPY5 stabilizes its retention on the fibers, preventing leaching during subsequent use. From a functional perspective, the subtle molecular mobility of these non-covalently bound compounds enhances contact with microbial cells. Since reactive oxygen species (ROS), such as singlet oxygen, have short half-lives and operate over nanometer distances, this proximity is critical for maximizing the efficiency of photodynamic inactivation. The primary advantages of this method lie in its technological simplicity and cost-effectiveness.

### 2.4. Generation of Reactive Oxygen (^1^O_2_) from BODIPY5 and Cotton Fabric Treated with It

The generation of reactive oxygen species from BODIPY5 in solution and after immobilization on textile was monitored by measuring the photooxidation of potassium iodide (KI), a widely used indicator of photoinduced oxidative processes. This method is based on the oxidation of iodide anions (I^−^) to molecular iodine (I_2_), which, in the presence of excess iodide, forms the triiodide anion (I_3_^−^) [50,51]. The process can be followed by UV–Vis spectroscopy due to the characteristic absorption band of I_3_^−^ at 352 nm.

**Figure 2:**
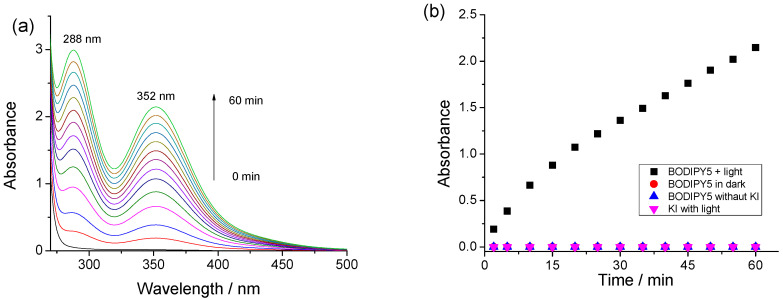
UV–vis absorption spectra indicating photoinduced oxidation of iodide to triiodide (I_3_^−^) in the presence of BODIPY5 under irradiation: (**a**) absorption spectra recorded at different irradiation times; (**b**) time-dependent changes in absorbance at 352 nm under different conditions: KI with light; BODIPY5 without KI; BODIPY5 in the dark; and BODIPY5 under irradiation.

Figure 2 shows the absorption spectra recorded in a homogeneous medium, where a progressive increase in the intensity of the band at 352 nm is observed with irradiation time, indicating the formation of I_3_^−^. No significant changes were detected under dark conditions or in the absence of KI, confirming that the observed process is light-driven and probe-dependent.

It should be noted that the iodide oxidation method is not fully selective for singlet oxygen and may also reflect the contribution of other reactive oxygen species. Therefore, the results are interpreted as evidence of photoinduced ROS generation rather than as unambiguous proof of a Type II mechanism. Nevertheless, the presence of iodine substituents in the BODIPY structure promotes efficient intersystem crossing via the heavy-atom effect, supporting the expected ability of BODIPY5 to generate singlet oxygen.

In homogeneous solution, the process is rapid and efficient due to the complete dissolution of the photosensitizer and unrestricted diffusion of molecular oxygen. The spectral data show a steep and continuous increase in I_3_^−^ absorbance at 352 nm during the initial stages of irradiation, reflecting efficient photoinduced oxidation processes.

**Figure 3:**
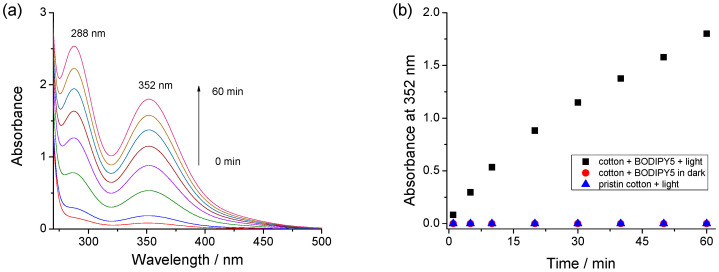
UV–vis absorption spectra showing the formation of triiodide (I_3_^−^) in the presence of cotton fabric treated with BODIPY5 under irradiation: (**a**) absorption spectra recorded at different irradiation times; (**b**) time-dependent changes in absorbance at 352 nm for pristine cotton under irradiation; BODIPY5-treated cotton in the dark; and BODIPY5-treated cotton under irradiation.

Figure 3 further demonstrates that non-covalently immobilized BODIPY5 on the cotton matrix retains its photodynamic activity on the solid substrate. Upon irradiation, an increase in absorbance at 352 nm is also observed for the textile system, indicating that reactive oxygen species generated at or near the fiber surface can diffuse into the surrounding medium. In contrast, untreated cotton fabric does not show any spectral changes under the same conditions, confirming that the observed activity originates from the immobilized iodinated BODIPY derivative.

A difference in the process kinetics is observed for the cotton-supported system. Although BODIPY5 immobilized on the fibers retains its ability to generate reactive oxygen species, the overall signal intensity measured in the surrounding solution is lower compared to that obtained in homogeneous solution. This behavior can be attributed to partial aggregation of the chromophores and limited diffusion of oxygen and reactive species within the textile matrix.

The main difference between the homogeneous and heterogeneous systems lies in the spatial localization of the process. In solution, the reaction occurs uniformly throughout the volume, whereas on the textile substrate it is localized at the surface. This surface-confined activity is particularly advantageous for antimicrobial applications, as it enables the generation of reactive oxygen species directly at the interface where microorganisms interact with the material.

Overall, the results demonstrate that, despite the transition from a homogeneous to a heterogeneous environment, the photophysical behavior of BODIPY5 remains effective, allowing cotton fabric to function as a light-activated antimicrobial material.

### 2.5. Antimicrobial Activity of BODIPY5 in Homogeneous Medium and on Cotton Fabric

**Figure 4:**
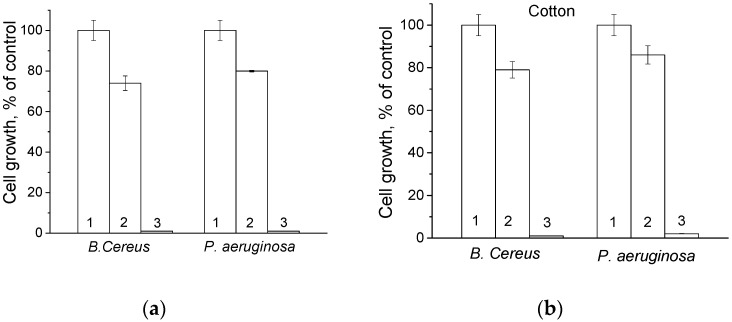
Comparative analysis of the viability of *B. cereus* and *P. aeruginosa* after treatment with BODIPY5 (10 µg/mL) in homogeneous medium (**a**) and on functionalized cotton fabric (**b**). The experimental conditions are defined as follows: (1) control—bacterial suspension without a photosensitizer, kept in the dark; (2) bacteria in the presence of BODIPY5 (or BODIPY5-treated cotton) under dark conditions; (3) bacteria in the presence of BODIPY5 (or BODIPY5-treated cotton) after irradiation with visible light.

The photodynamic activity of BODIPY5 against the model bacterial strains *B. cereus* and *P. aeruginosa* is shown in Figure 4, with cell growth compared under dark and irradiated conditions. The control group establishes the baseline level of cell viability, confirming that in the absence of a photosensitizer and light irradiation, bacterial cultures develop normally. The experimental conditions are defined as follows: control (bacteria without a photosensitizer, kept in the dark), dark (bacteria in the presence of BODIPY5 without irradiation), and irradiated samples (bacteria in the presence of BODIPY5 after light exposure).

When bacteria are treated with BODIPY5 at 10 µg/mL in the dark, moderate growth inhibition is observed. For the Gram-positive *B. cereus*, viability is reduced to approximately 74%, while for the Gram-negative *P. aeruginosa*, it remains around 80%. This “dark toxicity” indicates that the iodinated BODIPY core possesses some intrinsic antimicrobial activity, likely due to lipophilic interactions with bacterial membranes. However, the relatively high cell survival under these conditions suggests that the compound alone is not sufficiently toxic in the absence of light.

Upon irradiation with visible light, a dramatic decrease in cell growth is observed, reaching levels below 1%, indicating nearly complete eradication of bacterial colonies. The antibacterial activity of BODIPY5 was further evaluated over a concentration range of 10–50 µg/mL. Under dark conditions, a relatively weak concentration-dependent effect is observed, with increasing inhibition at higher concentrations. In contrast, upon light irradiation, a very strong photodynamic effect is already achieved at the lowest concentration tested (10 µg/mL), resulting in >99% inhibition of bacterial growth. At higher concentrations, the activity remains at similarly high levels (>99%), indicating a saturation (plateau) effect under the conditions tested.

This pronounced effect is a direct consequence of the structural modification of the fluorophore with two iodine atoms, which induces efficient intersystem crossing (ISC), redirecting the energy from the excited singlet state to the triplet state. This enables the molecule to interact with molecular oxygen and generate singlet oxygen and other reactive oxygen species.

The high efficacy of BODIPY5 against the Gram-negative bacterium *P. aeruginosa* deserves special attention. Due to their complex outer membrane, Gram-negative bacteria are usually highly resistant to photodynamic agents. The achieved almost complete cell killing at a low concentration of 10 µg/mL proves that the singlet oxygen generated by BODIPY5 is in sufficient quantity and with sufficient potency to overcome this biological barrier. This indicates that BODIPY5 functions as a highly effective light-activated antibacterial agent with minimal toxicity in the absence of light.

The putative mechanism of BODIPY5’s antibacterial activity is based on the principles of aPDI, driven by the specific photophysical properties of the modified core [52]. The main factor for the high efficacy of the compound is the introduction of two iodine atoms, which induce the so-called “heavy atom effect”. Upon irradiation with visible light, the molecule absorbs photons and transitions to an excited singlet state (S1). Instead of releasing this energy through fluorescence, the presence of the iodine substituents dramatically enhances intersystem crossing (ISC), directing the molecule to the more stable and reactive triplet state (T_1_). From this triplet state, BODIPY5 acts as a potent photosensitizer, transferring its energy directly to molecular oxygen (^3^O_2_) present in the cellular environment. This process generates singlet oxygen (^1^O_2_), an extremely reactive oxygen species. Due to its lipophilic nature, BODIPY5 has a high affinity for bacterial lipid membranes, where it localizes prior to irradiation. In situ-generated singlet oxygen triggers massive lipid peroxidation and oxidative damage to membrane proteins.

This leads to irreversible disruption of the bacterial envelope, loss of osmotic control, and subsequent cell death. This mechanism is equally effective against Gram-positive bacteria and the more complex envelopes of Gram-negative strains such as *P. aeruginosa*, as ROS act through non-specific oxidation, which prevents the development of bacterial resistance. The results in Figure 4 confirm this model, demonstrating that the potent bactericidal effect is tightly controlled by light, making BODIPY5 a precise therapeutic tool.

Comparing the activity of BODIPY5 in solution and on cotton fabric shows a high degree of correlation in the mechanism of action of the photosensitizer, regardless of its physical form. When measuring bacterial viability in the dark, it was observed that immobilizing the photosensitizer on cotton fibers resulted in a slight increase in survival (about 5–6%) compared to the solution (Figure 4). This is likely due to the lower bioavailability of BODIPY5 when bound to the cotton matrix, limiting its direct contact with bacterial membranes in the absence of light. In both cases, minimal “dark” activity was observed, with cell growth of *B. cereus* and *P. aeruginosa* maintained at 75–85%. This confirms that immobilization of BODIPY5 on cotton fibers does not change its low intrinsic toxicity, which is essential for the safety of biomedical materials in the absence of light.

When activated with light, the cotton fabric exhibits pronounced self-disinfection properties. The viability of the bacteria drops to levels below 1–2%, which is fully comparable to the results obtained in solution, proving that the generated singlet oxygen successfully diffuses from the surface of the cotton fibers to the attached bacterial cells. This indicates that BODIPY5 retains its ability to generate singlet oxygen when adsorbed onto cellulose fibers.

The comparison with the Gram-negative strain *P. aeruginosa* is particularly important. Although the cotton fabric has a heterogeneous surface that could limit the diffusion of reactive oxygen species (ROSs), the results show almost complete inactivation of pathogenic bacteria. This indicates a high local concentration of singlet oxygen at the fiber surface, sufficient to overcome the complex cell wall of Gram-negative bacteria. Thus, the functionalized textile does not merely passively inhibit bacterial growth but actively self-disinfects under ambient light. This is direct evidence of the successful development of a “smart” textile surface. By using iodinated BODIPY, a transition from passive protection to active photoinduced disinfection was achieved. The achieved efficacy against the Gram-negative model *P. aeruginosa* on cotton fabric highlights the potential of this material for application in hospital environments, where surface contamination is a major source of infections.

### 2.6. SEM Analysis of Cotton Fabrics

**Figure 5:**
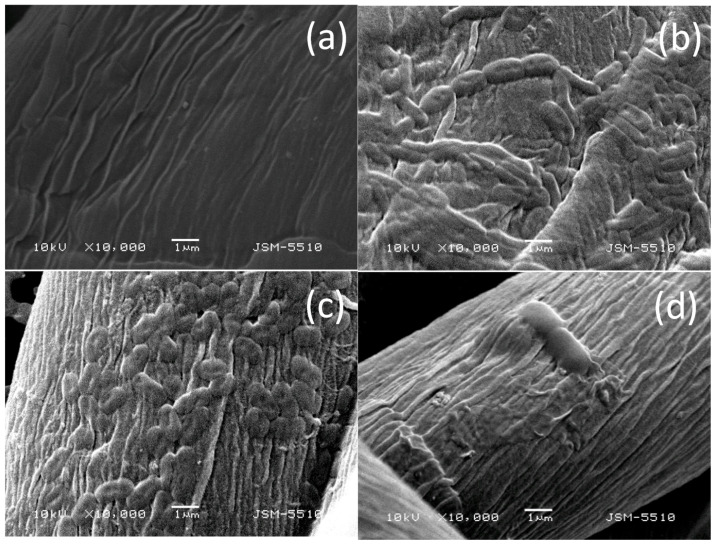
SEM micrographs (magnification x10,000) of the surface of cotton fabric incubated with *P. aeruginosa*: (**a**) untreated cotton fabric (control); (**b**) colonization of bacterial cells on untreated fabric; (**c**) BODIPY5-modified fabric under dark conditions; (**d**) photoinduced inactivation and deformation of bacterial cells on modified fabric after irradiation. Scale bar corresponds to 1 μm.

Figure 5 shows a scanning electron microscopy image of cotton fabric at a magnification of x10,000, which visualizes the surface morphology and interaction with *P. aeruginosa* bacterial cells. The micrograph in Figure 5a shows the surface of an untreated cotton fabric, which was used as a control, in which the characteristic longitudinal fibrillar structure of the cellulose fibers is visible. When the untreated fabric was exposed to a bacterial suspension (as shown in the micrograph in Figure 5b), massive colonization of the surface by *P. aeruginosa* was observed, with the bacteria exhibiting their typical rod shape and tightly adhering to the fibers, indicating unhindered biofilm formation on the untreated textile. After treating the fabric with BODIPY5 under dark activation conditions in the presence of bacteria, a certain amount of attached bacterial cells was observed in micrograph shown in Figure 5c, but their number was reduced compared to the control, and the clusters were less pronounced, suggesting a possible weak dark cytotoxicity. Upon irradiation of the treated fabric with sunlight, a drastic reduction in the bacterial population was observed (as shown in the micrograph in Figure 5d). The remaining cells showed impaired morphology, loss of structural integrity, and signs of deformation, indicating the potent antibacterial activity of BODIPY5 induced by light through photodynamic inactivation, which does not simply retain microorganisms but actively destroys them upon exposure to light.

### 2.7. Comparison with Reported Photosensitizers

The results summarized in Table 2 show that 2,6-diiodinated BODIPY 5 exhibits significantly higher photoinduced antibacterial activity compared to several photosensitizers previously developed by us or reported in the literature. At a concentration of 10 µg/mL (≈17 µM), the compound achieved over 99% inhibition of microbial growth against both Bacillus cereus and Pseudomonas aeruginosa, indicating high efficacy against both Gram-positive and Gram-negative bacteria.

However, comparison with literature data for cationic iodinated BODIPY derivatives shows that similar antimicrobial activity can be achieved at lower concentrations (0.5–5 µM). Similarly, other functionalized cationic BODIPY compounds exhibit high efficacy (90–99%) in the range of 1–10 µM. In contrast, non-cationic (neutral) iodinated BODIPY derivatives typically require higher concentrations (approximately 5–10 µM) to achieve comparable effects (>99% reduction), which is consistent with their lower cellular association and more limited penetration into bacterial cells. An important factor contributing to the higher efficiency of cationic BODIPY derivatives is their positive charge, which facilitates electrostatic interactions with negatively charged bacterial cell walls. This results in enhanced accumulation of the photosensitizer within the cells, and consequently more efficient generation of reactive oxygen species in situ.

In comparison, some 1,8-naphthalimide-modified dendrimers show significantly lower photodynamic efficiency. For example, at similar or even higher concentrations (1.4–90 µM), these systems achieve 62–83% inhibition, with only first-generation PAMAM-NI reaching nearly 98% against *B. cereus*, but at a significantly higher concentration (~90 µM). In Gram-negative bacteria, the effect remains limited (up to ~50%). Nevertheless, it should be noted that these dendrimer systems exhibit relatively higher activity under dark conditions, which may represent an advantage in applications where light activation is limited or not feasible.

The results show that 2,6-diiodinated BODIPY 5 exhibits strong antibacterial photodynamic activity, comparable to some of the effective photosensitizers reported in the literature. It should be noted that direct comparison with literature data is challenging due to differences in experimental conditions, such as irradiation time, light intensity, and the bacterial strains used. An additional advantage of BODIPY 5 is its water insolubility, which allows stable deposition onto cotton fabric without leaching from the textile surface. Such an effect would be less likely for cationic BODIPY derivatives, which are generally more soluble and mobile in aqueous environments. This highlights the potential of BODIPY 5 for practical application in the development of self-disinfecting textile materials, with possible applications in medicine and protective coatings

## 3. Materials and Methods

### 3.1. Materials and Reagents

All reagents and solvents were purchased from Merck, Hamburg, Germany and used without further purification. The cotton fabric (100% cotton, 140 g/m^2^) was pre-washed with a non-ionic detergent to remove any residual sizing agents and dried at room temperature. NMR spectra were recorded on a Bruker Avance 300 or 500 spectrometer (Bruker, Ettilingen, Germany)at ambient temperature in CDCl_3_ as the solvent and tetramethylsilane (TMS) as the reference. Reaction monitoring was performed via thin-layer chromatography (TLC) on silica gel-coated aluminum plates (POLYGRAM^®^ SIL G/UV254, Merck), with detection under UV illumination. The model bacterial strains *Bacillus cereus* (Gram-positive) and *Pseudomonas aeruginosa* (Gram-negative) were obtained from the National Bank for Industrial Microorganisms and Cell Cultures (NBIMCC), Bulgaria.

### 3.2. Synthesis of p-(tert-butyldimethylsilyloxy)benzaldehyde

A solution of *tert*-butyldimethylsilyl chloride (0.55 g, 3.2 mmol) in 5 mL of dry DCM was added dropwise to *p*-hydroxybenzalehyde (0.3 g, 2.46 mmol) and Et_3_N (0.5 mL, 3.1 mmol) dissolved in dry DCM (15 mL) followed by stirring the reaction mixture for 10 h. Then, the reaction mixture was diluted with H_2_O (30 mL) and extracted using DCM (3 × 20 mL). The combined DCM layers were dried over Na_2_SO_4_, filtered and concentrated under reduced pressure. Then, the residue was purified by column chromatography using hexane/EtOAc 10/1 as an eluent to obtain the title compound as a pale-yellow oil (0.52 g, 88%). ^1^H NMR (500 MHz, CDCl_3_) δ 9.8 (s, 1H), 7.7 (d, *J* = 8.5 Hz, 2H), 6.9 (d, *J* = 8.5 Hz, 2H), 0.9 (s, 9H), 0.2 (s, 6H).

### 3.3. Synthesis of Meso-(tert-butyldimethyl(phenoxy)silane)-Appended BODIPY**3**

To a mixture of 2,4-dimethylpyrrole (2.3 mmol, 0.23 g) and *p*-(*tert*-butyldimethylsilyloxy) benzaldehyde (1.0 mmol, 0.23 g) dissolved in dried and degassed DCM (15 mL) under Ar protection, two drops of trifluoroacetic acid (TFA) were added and the mixture was stirred at room temperature for 10 h. Then, a solution of 2,3-dichloro-5,6-dicyano-1,4-benzoquinone (DDQ) (1.0 mmol, 0.23 g) in 5 mL of DCM was added, followed by continued stirring for 1 h. Then, 3 mL of TEA were added, followed by the addition of 3 mL of boron trifluoride diethyl etherate (BF_3_OEt_2_). After 12 h of stirring at RT, the solvent was evaporated under reduced pressure, and the residue was purified by column chromatography using EtOAc/hexane (1:10) as an eluent to afford a red solid product. Yield: 0.34 g (33%). ^1^H NMR (300 MHz, CDCl_3_) δ 6.8 (d, *J* = 8.4 Hz, 4H); δ 6.90 (d, *J* = 8.4 Hz, 2H); 6.8 (d, *J* = 8.4 Hz, 2H); 5.8 (s, 2H); 2.3 (s, 6H); 1.2 (s, 6H); 0.8 (s, 9H). ^13^C NMR (126 MHz, CDCl_3_) δ 156.5, 155.3, 143.1, 141.9, 131.8, 129.2, 128.0, 121.1, 25.7, 18.3, 14.6, 14.4, −4.4. ESI-TOF mass spectrum 455.25 (57%, M^+^ + 1).

### 3.4. Synthesis of 2,6-Diiodo-Substituted Meso-(tert-butyldimethyl(phenoxy)silane)-Appended BODIPY**4**

To a solution of BODIPY**3** (0.1 g, 0.22 mmol) in dried DCM/(20 mL), *N*-iodosuccinimide (0.24 g, 1.1 mmol) was added, followed by stirring in the dark for 10 h at ambient temperature. Then, the red solution was washed using H_2_O (50 mL × 2) followed by drying over anhydrous Na_2_SO_4_ and concentrating under reduced pressure; then, the residue was purified by column chromatography using hexane/EtOAc (10:1, *v*/*v*) to afford the title compound as a red solid (0.06 g, 68%). ^1^H NMR (500 MHz, CDCl_3_); δ 7.1 (d, *J* = 8.0 Hz, 2H); 7.0 (d, *J* = 8.0 Hz, 2H); 2.7 (s, 6H); 1.5 (s, 6H); 1.0 (s, 9H). ^13^C NMR (126 MHz, CDCl_3_) δ 156.0, 155.6, 144.3, 140.6, 130.6, 128.1, 126.6, 120.4, 84.5, 24.7, 17.4, 16.0, 15.0, −5.5. ESI-TOF mass spectrum 678.0 (100%, M^+^).

### 3.5. Synthesis of 2,6-Diiodo-Substituted Meso-(p-hydroxyphenyl)-Appended BODIPY**5**

To a solution of 100 mg of 2,6-diiodo-substituted meso-(tert-butyldimethyl(phenoxy)silane)-appended BODIPY**4**, in a 20 mL mixture of DCM/methanol (1:1), 100 µL TEA were added, followed by refluxing until the reaction was complete. Then, the solvent was evaporated under reduced pressure, and the residue was purified by column chromatography using n-hexane/ethyl acetate (10:1–2:1) to obtain the pure red product (0.075, 90%). ^1^H NMR (500 MHz, CDCl_3_) δ 7.0 (d, *J* = 8.3 Hz, 2H); 6.9 (d, *J* = 8.3 Hz, 2H); 5.1 (s, 1H); 2.6 (s, 6H); 1.4 (d, *J* = 10.6 Hz, 6H). ^13^C NMR (75 MHz, CDCl_3_) δ 162.2, 160.2, 149.5, 146.3, 135.7, 132.9, 129.3, 120.2, 20.9, 19.7. ESI-TOF mass spectrum 572.95 (100%, M^+^ − F).

### 3.6. Iodometric Measurements

BODIPY5 (c = 1 × 10^−6^ M) was added to 20 mL of an aqueous solution of KI (0.5 M), and the solution was illuminated with sunlight using a Newport solar simulator (150W Xe, 36 mW cm^−2^) for 60 min. A 1 cm^−2^ piece of cotton fabric treated with BODIPY5 was placed in 20 mL of KI solution (c = 0.5 M), and the system was irradiated in a manner similar to the BODIPY5 solution. The samples were positioned at a distance of 25 cm from the light source. Absorption spectra in the range of 250 to 500 nm were recorded for the irradiated aqueous solutions.

### 3.7. Scanning Electron Microscopy (SEM)

SEM was employed to evaluate bacterial adhesion and biofilm development on the cotton substrate. Specifically, tubes containing MPB and specimens of both untreated and treated fabrics were inoculated with a *P. aeruginosa* suspension. After a 24 h incubation period, the materials were rinsed with phosphate-buffered saline and dried. To prepare for imaging, they were gold-coated using a JFC-1200 fine coater (Jeol Ltd., Tokyo, Japan). Finally, the surface morphology was examined at various magnifications using a JSM-5510 scanning electron microscope from the same manufacturer.

### 3.8. Treatment of Cotton Fabric with BODIPY5

BODIPY5 (5 mg) was dissolved in 1 mL of ethanol, and the resulting solution was added to 20 mL of deionized water. A total of 1 g of cotton fabric was immersed in the prepared fine emulsion, and the temperature was maintained at 50 °C for 2 h under constant stirring. Subsequently, the fabric was removed, air-dried at room temperature, rinsed with water, and dried again. The amount of deposited BODIPY5 was determined by measuring the fluorescence intensity of the solutions before and after the treatment process. It was found that the dye adsorbs efficiently onto the cotton surface, with 4.38 mg (88% of the initial amount) being successfully deposited per 1 g of cotton fabric.

### 3.9. Evaluation of BODIPY5 in Solution

The antibacterial efficacy of BODIPY5 was evaluated against two model strains: Gram-positive *Bacillus cereus* and Gram-negative *Pseudomonas aeruginosa* (obtained from the Collection of the Institute of Microbiology, Bulgarian Academy of Sciences). Growth inhibition was assessed in meat-peptone broth (MPB) under both dark and light-exposed conditions. Stock solutions were prepared in DMSO at an initial concentration of 1.0 mg/mL and subsequently diluted in MPB to achieve a range of final concentrations (50, 40, 25, 15, and 10 µg/mL). Duplicate sets of test tubes were inoculated with standardized microbial suspensions (adjusted to an initial optical density at 600 nm (OD_600_) of approximately 0.1) and incubated for 24 h at the appropriate temperature under constant agitation (240 rpm). Microbial proliferation was quantified by measuring the optical density at 600 nm. All experiments were performed in triplicate, and the results are presented as mean values (standard deviation <5%).

### 3.10. Antimicrobial Performance of Functionalized Cotton

The activity of the BODIPY5-treated cotton was similarly tested against the same bacterial models. Square-shaped fabric specimens (1 cm—1 cm) were immersed in tubes containing MPB and inoculated with the respective bacterial suspensions. To ensure accuracy, several controls were included: untreated cotton specimens; blank controls (MPB with inoculum only); and sterility controls (compounds in MPB without inoculum). Following a 24 h incubation period under either light irradiation or in darkness, the fabric samples were removed, and the OD_600_ of the remaining medium was recorded to determine the level of growth inhibition. All measurements were conducted in triplicate, and the averages were taken (standard deviations less than 5%).

## 4. Conclusions

The present study describes the synthesis and characterization of a novel iodinated BODIPY5 derivative and its effective immobilization on cotton fabric to create an active, self-disinfecting surface. By introducing two iodine atoms into the photosensitizer’s structure, a strong “heavy atom effect” was achieved, optimizing singlet oxygen generation and providing good antibacterial activity upon visible-light irradiation. The potential of BODIPY5 as a highly effective antimicrobial photodynamic therapy (aPDT) agent was demonstrated, both in a homogeneous medium and when deposited on cotton fabric. While untreated cotton fabric is highly susceptible to bacterial colonization and biofilm formation, the functionalized material can almost completely eliminate pathogenic microorganisms such as *P. aeruginosa* and *B. cereus*. It has been shown that the antimicrobial efficacy is preserved when BODIPY5 is immobilized on cotton fabric. While in the dark, bacterial viability remains relatively high (~74–86%) and light activation leads to almost complete eradication (growth <2%) of both model strains—Gram-positive *B. cereus* and the highly resistant Gram-negative *P. aeruginosa*. The ability of the treated cotton fabric to suppress the growth of *P. aeruginosa* to nearly 2% is a particularly significant achievement, demonstrating that surface-generated ROSs are powerful enough to overcome the complex cellular barriers of these pathogens. The resulting textile material possesses pronounced self-disinfecting properties activated by visible light, making it highly suitable for applications such as protective hospital clothing and biomedical dressings. This approach offers a sustainable alternative to traditional antibiotics, minimizing the risk of bacterial resistance development through non-specific photodynamic oxidation. Although the reusability of the treated cotton fabric was not investigated in the present study, the water-insoluble nature of BODIPY5 suggests strong retention on the textile surface after deposition, likely supported by hydrogen-bonding interactions with the cellulose matrix. This feature is expected to reduce the likelihood of photosensitizer leaching and enhance the stability of the functionalized material during use. Nevertheless, further studies are required to evaluate the washing durability and long-term reusability of the textile under practical conditions.

## Figures and Tables

**Table 1:** Photophysical characteristics of BODIPY3, BODIPY4, BODIPY5 in organic solvents. Standard used was rhodamine 6G in EtOH, R6G Φ ≈ 0.95.

	DMSO	Ethanol	Dioxane	CH_3_CN	CH_2_Cl_2_
**BODIPY3**					
λ_abs._ (nm)	501	499	501	497	501
λ_fl_. (nm)	521	517	520	516	519
Stokes shift (cm^−1^)	766	698	729	741	692
ε (M^−1^cm^−1^)	43,312	40,384	59,595	28,686	67,273
*Φ_F_*	0.515	0.373	0.446	0.304	0.377
**BODIPY4**					
λ_abs._ (nm)	535	531	533	528	533
λ_fl_. (nm)	561	557	554	548	554
Stokes shift (cm^−1^)	866	879	711	691	711
ε (M^−1^cm^−1^)	59,111	61,295	35,553	61,173	56,058
*Φ_F_*	0.012	0.017	n.d.	0.013	0.022
**BODIPY5**					
λ_abs._ (nm)	534	529	532	527	529
λ_fl_. (nm)	555	554	556	548	556
Stokes shift (cm^−1^)	709	853	811	727	776
ε (M^−1^cm^−1^)	56,324	47,173	52,239	75,000	34,742
*Φ_F_*	0.015	0.012	n.d.	0.009	0.018

**Table 2:** Comparison of the photodynamic antibacterial activity of BODIPY 5 with previously reported photosensitizers.

	Photosensitizer	Microorganisms Tested	Photosensitizer Concentration	Microbial Growth Reduction	Reference
1	2,6-diiodo BODIPY 5	*B. cereus*, *P. aeruginosa*	16 µM	>99%	(This Work)
	PAMAM-NI-3rd-generation	*B. cereus*, *P. aeruginosa*	1.4 µM	78% (*B. cereus*) 62% (*P. aeruginosa*)	[53]
2	PAMAM-NI-2nd-generation	*B. cereus*, *P. aeruginosa*	6.8 µM	83% (*B. cereus*) 75% (*P. aeruginosa*)	[54]
3	PAMAM-NI-1st-generation	*B. cereus*, *P. aeruginosa*	90 µM	~98% (*B. cereus*) 50% (*P. aeruginosa*)	[44]
4	Cationic iodinated BODIPY	*S. aureus*, *E. coli*	0.5–5 µM	99.9%	[40]
5	Functionalized BODIPY (cationic groups)	*E. coli*, *S. aureus*	1–10 µM	90–99%	[55]
6	Neutral iodinated BODIPY (lipophilic derivative)	*S. aureus*	5–10 µM	>99%	[56]

## Data Availability

The original contributions presented in this study are included in the article/Supplementary Materials. Further inquiries can be directed to the corresponding authors.

## Supplementary Materials

The following supporting information can be downloaded at: https://www.mdpi.com/article/10.3390/molecules31091525/s1, Figure S1: ^1^H-NMR spectrum of **2**, Figure S2. ^1^H-NMR spectrum of BODIPY**3**, Figure S3. ^13^C-NMR spectrum of BODIPY**3**, Figure S4. ESI mass spectrum of BODIPY**3**, Figure S5. ^1^H-NMR spectrum of the monomer BODIPY**4**, Figure S6. ^13^C-NMR spectrum of the monomer BODIPY**4**, Figure S7. ESI mass spectrum of BODIPY**4**, Figure S8. ^1^H-NMR spectrum of BODIPY**5**, Figure S9. ^13^C-NMR spectrum of the monomer BODIPY**5**, Figure S10. ESI mass spectrum of BODIPY**5**.
