# Supplementary material for: Photoinduced Inactivation of Pathogenic Microorganisms via Cotton Textile Functionalized with a Novel Iodinated BODIPY Derivative
Source: Molecules. 2026 May 4;31(9):1525. doi: 10.3390/molecules31091525 (PMC13165066; doi:10.3390/molecules31091525)
Supplement: Supplementary file 1 [file molecules-31-01525-s001.zip › molecules-4254619-supplementary.pdf]

Supplementary Information

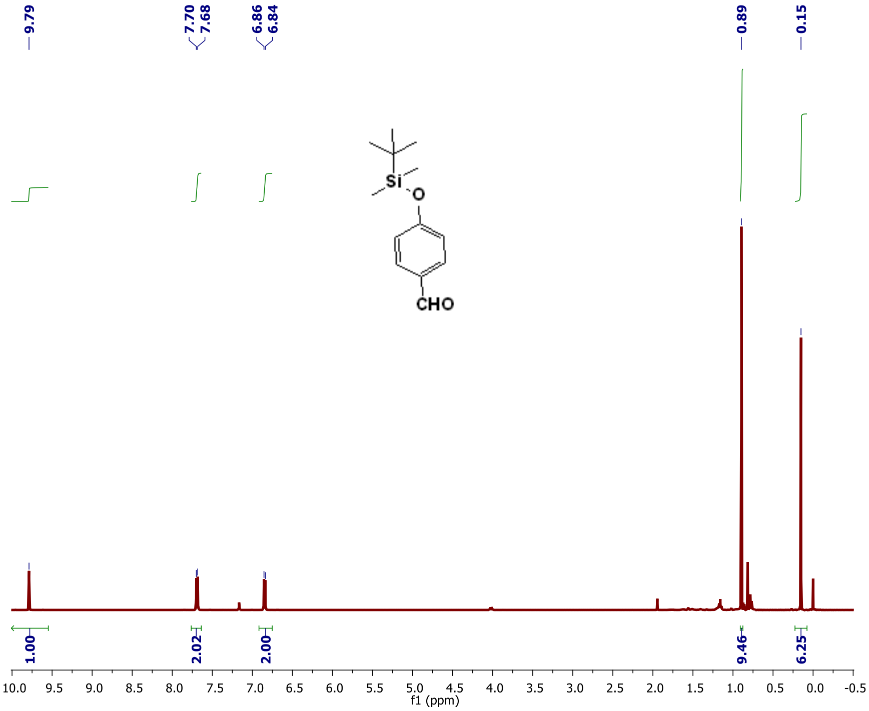

	Parameter	Value
1	Data File Name	D:/ AS_R_1/ 1/ pdata/ 1/ 1r
2	Title	AS_R_1
3	Comment	AS_R_1
4	Origin	Bruker BioSpin GmbH
5	Owner	nmr
6	Site	
7	Spectrometer	Avance
8	Author	
9	Solvent	CDCl3
10	Temperature	298.0
11	Pulse Sequence	zg30
12	Number of Scans	16
13	Receiver Gain	101
14	Relaxation Delay	1.5000
15	Pulse Width	8.0000
16	Acquisition Time	0.9999
17	Acquisition Date	2023-02-21 T22:49:07
18	Modification Date	2023-02-21 T19:49:08
19	Spectrometer Frequency	500.06
20	Spectral Width	9090.9
21	Lowest Frequency	-1516.8
22	Nucleus	1H
23	Acquired Size	9090
24	Spectral Size	65536

Figure S1. ^{1}H-NMR spectrum of **2**.

AS-2-proton

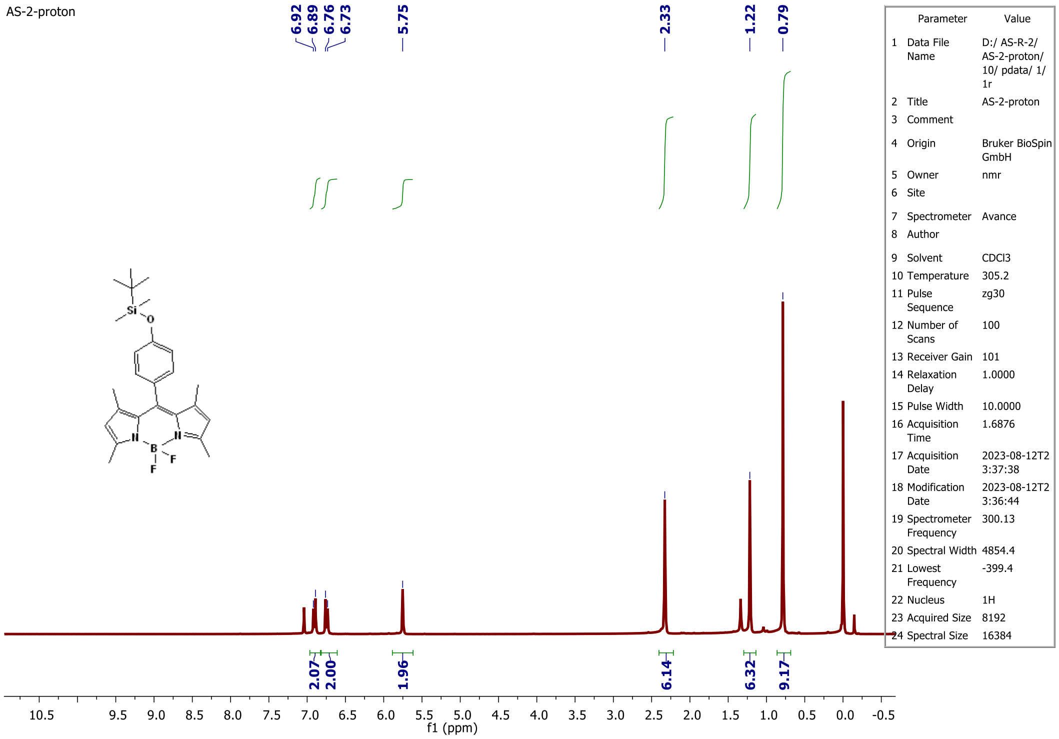

	Parameter	Value
1	Data File Name	D:/ AS-R-2/ AS-2-proton/ 10/ pdata/ 1/ 1r
2	Title	AS-2-proton
3	Comment	
4	Origin	Bruker BioSpin GmbH
5	Owner	nmr
6	Site	
7	Spectrometer	Avance
8	Author	
9	Solvent	CDCl3
10	Temperature	305.2
11	Pulse Sequence	zg30
12	Number of Scans	100
13	Receiver Gain	101
14	Relaxation Delay	1.0000
15	Pulse Width	10.0000
16	Acquisition Time	1.6876
17	Acquisition Date	2023-08-12T2 3:37:38
18	Modification Date	2023-08-12T2 3:36:44
19	Spectrometer Frequency	300.13
20	Spectral Width	4854.4
21	Lowest Frequency	-399.4
22	Nucleus	1H
23	Acquired Size	8192
24	Spectral Size	16384

Figure S2. ^{1}H-NMR spectrum of BODIPY**3**.

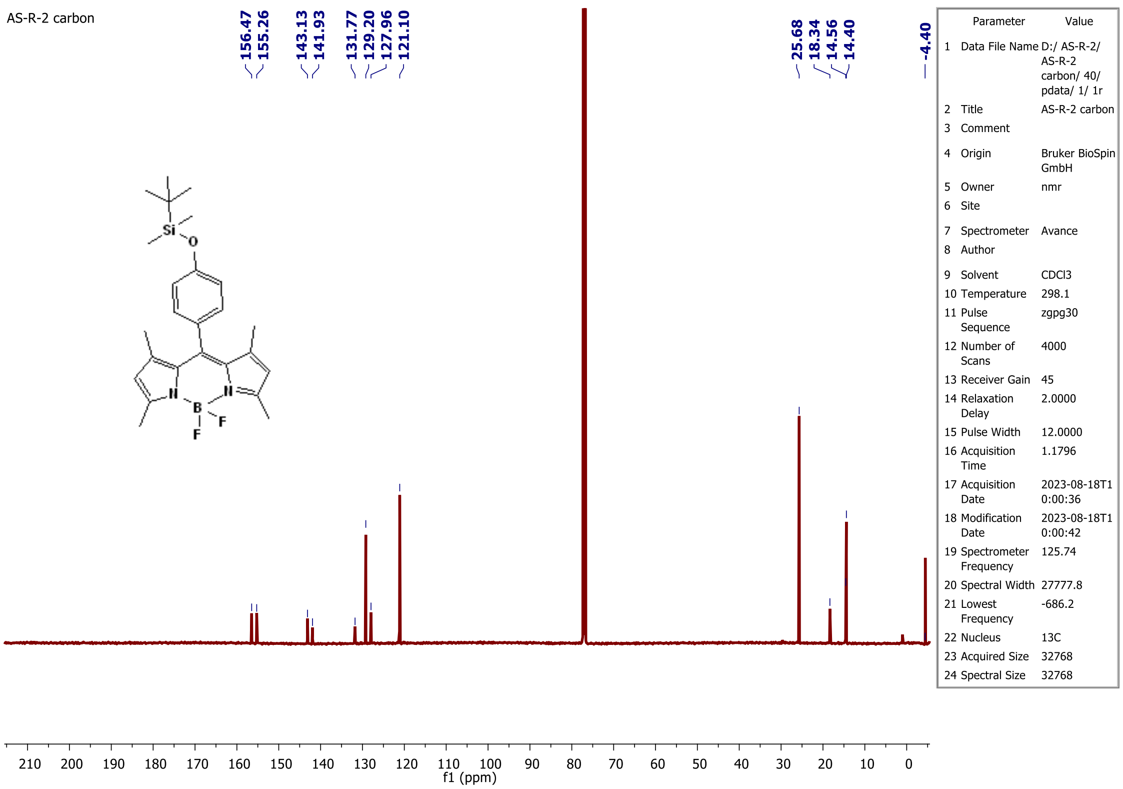

Figure S3. ^{13}C-NMR spectrum of BODIPY**3**.

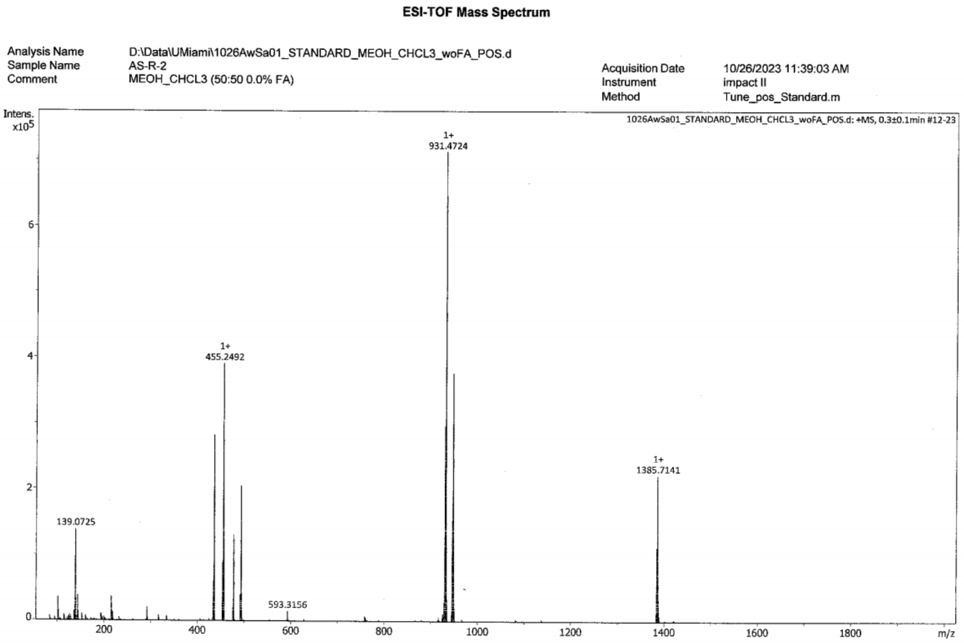

Figure S4. ESI mass spectrum of BODIPY**3**.

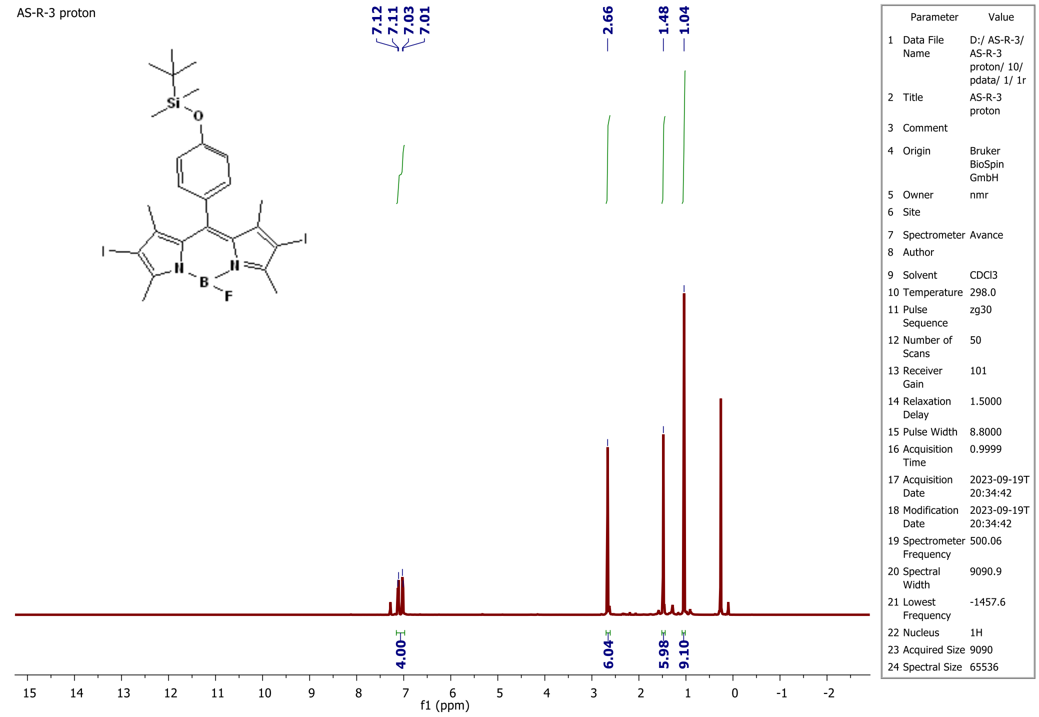

Figure S5. ^{1}H-NMR spectrum of the monomer BODIPY**4**.

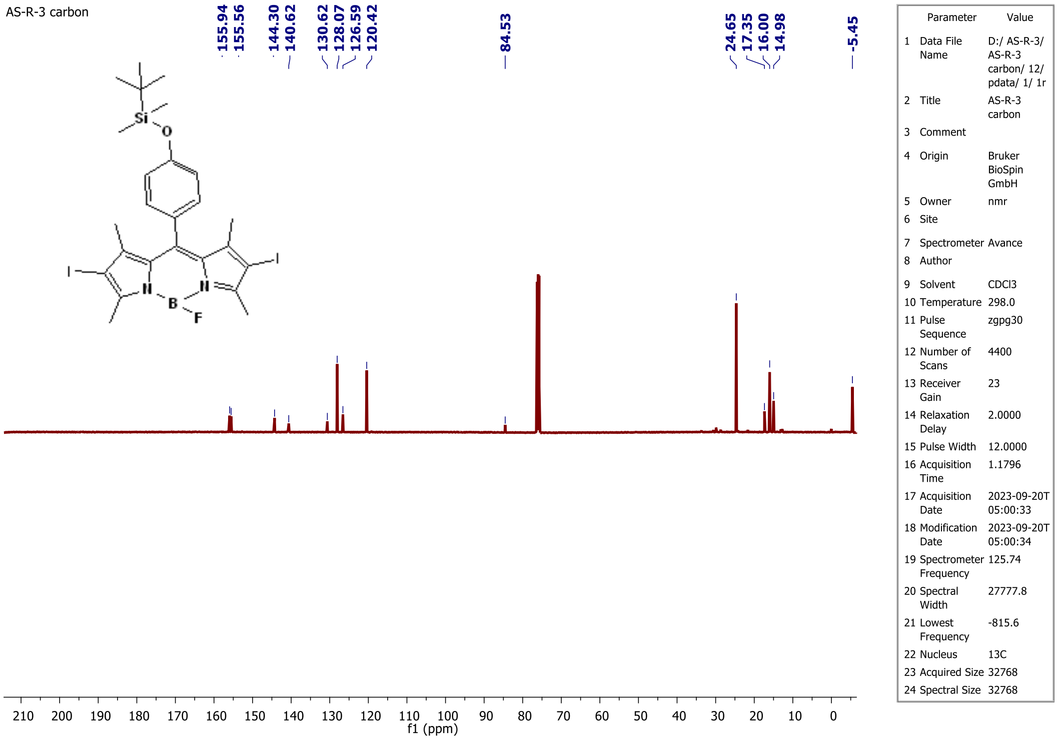

Figure S6. ^{13}C-NMR spectrum of the monomer BODIPY**4**.

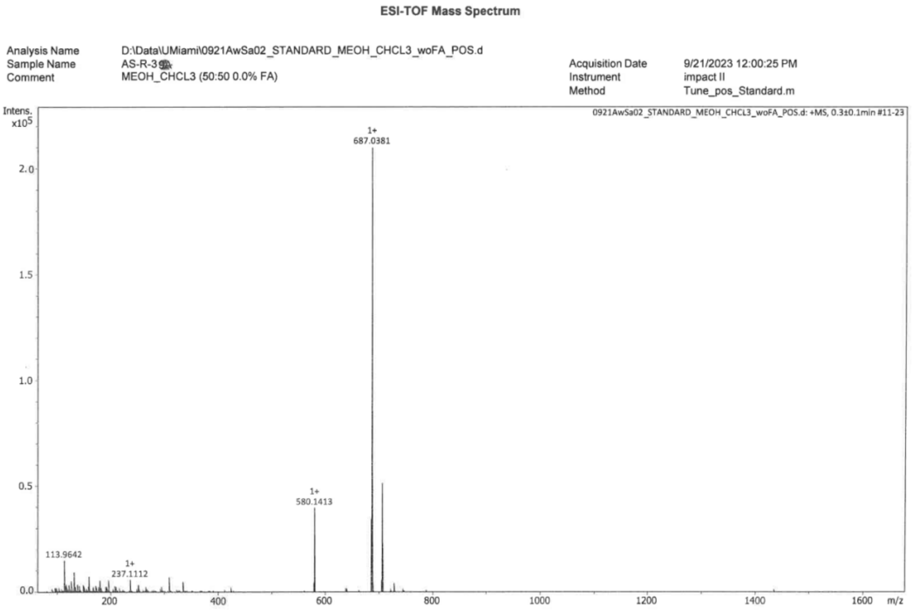

Figure S7. ESI mass spectrum of BODIPY**4**.

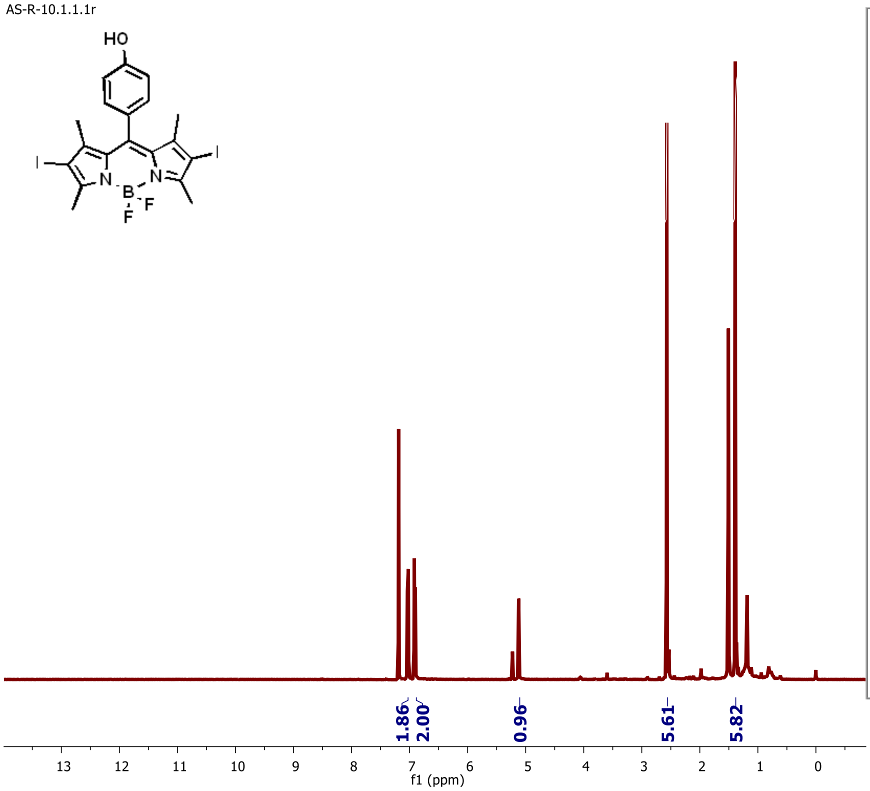

	Parameter	Value
1	Data File Name	D:/ prof. Ramyo francisco/ Francisco Ramyo/ NMR/ AS-R-10/ 1/ pdata/ 1/ 1r
2	Title	AS-R-10.1.1.1r
3	Comment	
4	Origin	Bruker BioSpin GmbH
5	Owner	nmr
6	Site	
7	Instrument	Avance
8	Author	
9	Solvent	CDCl3
10	Temperature	298.1
11	Pulse Sequence	zg30
12	Experiment	1D
13	Probe	Z44881_0025 (CP TCI 500S2 H-C/ N-D-05 Z)
14	Number of Scans	16
15	Receiver Gain	101.0
16	Relaxation Delay	1.5000
17	Pulse Width	8.0000
18	Presaturation Frequency	
19	Acquisition Time	0.9999
20	Acquisition Date	2023-05-15T11:12:52
21	Modification Date	2023-05-15T11:11:54
22	Class	
23	Spectrometer Frequency	500.06
24	Spectral Width	9090.9
25	Lowest Frequency	-1505.1
26	Nucleus	1H
27	Acquired Size	9090
28	Spectral Size	65536
29	Digital Resolution	0.14

Figure S8. ^{1}H-NMR spectrum of BODIPY**5**.

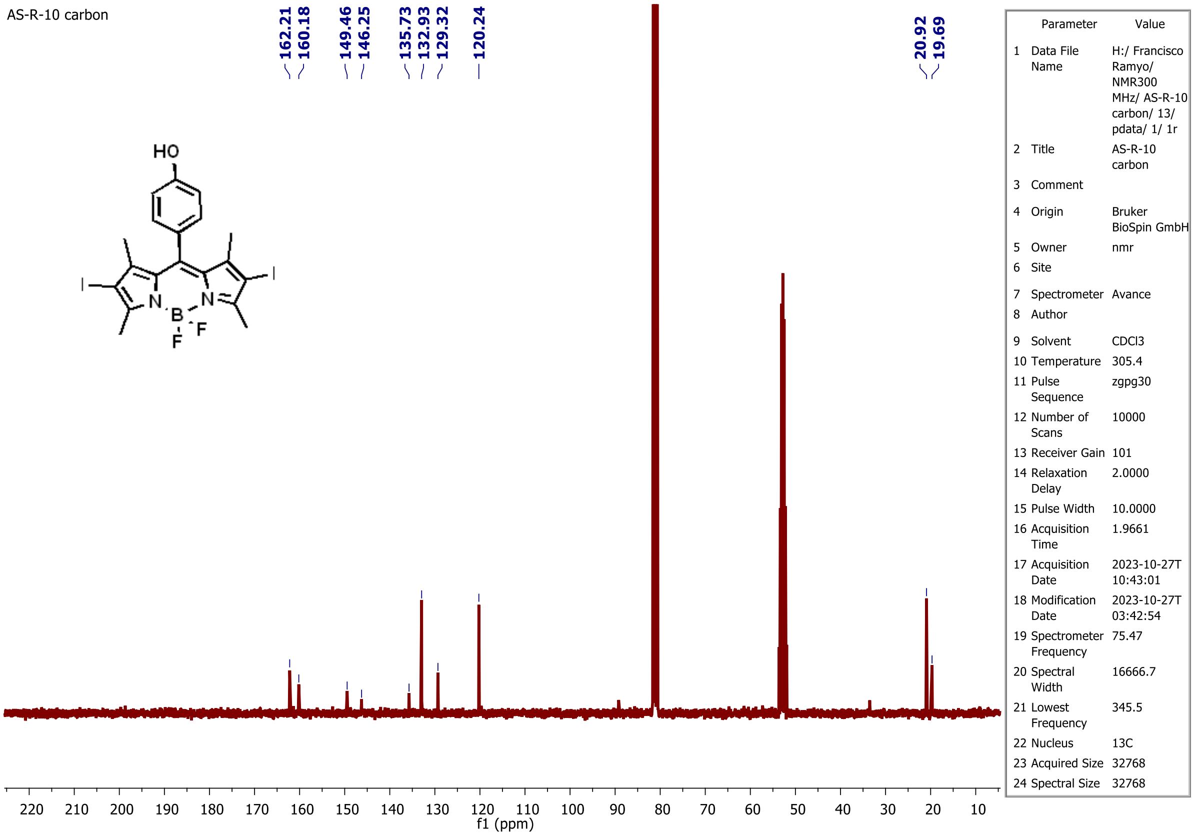

	Parameter	Value
1	Data File Name	H:/ Francisco Ramyo/ NMR300 MHz/ AS-R-10 carbon/ 13/ pdata/ 1/ 1r
2	Title	AS-R-10 carbon
3	Comment	
4	Origin	Bruker BioSpin GmbH
5	Owner	nmr
6	Site	
7	Spectrometer	Avance
8	Author	
9	Solvent	CDCl3
10	Temperature	305.4
11	Pulse Sequence	zgpg30
12	Number of Scans	10000
13	Receiver Gain	101
14	Relaxation Delay	2.0000
15	Pulse Width	10.0000
16	Acquisition Time	1.9661
17	Acquisition Date	2023-10-27T 10:43:01
18	Modification Date	2023-10-27T 03:42:54
19	Spectrometer Frequency	75.47
20	Spectral Width	16666.7
21	Lowest Frequency	345.5
22	Nucleus	13C
23	Acquired Size	32768
24	Spectral Size	32768

Figure S9. ^{13}C-NMR spectrum of the monomer BODIPY**5**.

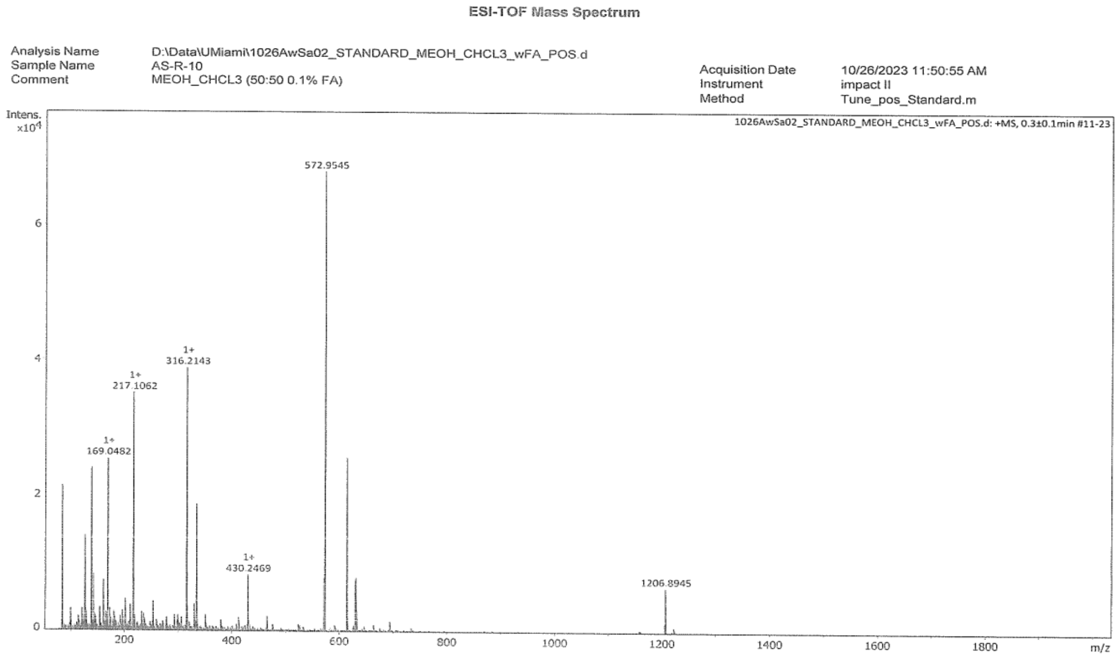

Figure S10. ESI mass spectrum of BODIPY**5**.
